# Comprehensive review of current management guidelines of chronic kidney disease

**DOI:** 10.1097/MD.0000000000033984

**Published:** 2023-06-09

**Authors:** Chukwuka Elendu, Rhoda C. Elendu, Joy M. Enyong, Jennifer O. Ibhiedu, Ibukunoluwa V. Ishola, Emmanuel O. Egbunu, Emmanuel S. Meribole, Sodiq O. Lawal, Chinazo J. Okenwa, Geraldine C. Okafor, Ewezugachukwu D. Umeh, Ozigis O. Mutalib, Kehinde A. Opashola, John O. Fatoye, Temitope I. Awotoye, Jewel I. Tobih-Ojeanelo, Habiba I. Ramon-Yusuf, Abiola Olanrewaju, Rechner N. Afuh, Joseph Adenikinju, Opeyemi Amosu, Abdulgafar Yusuf

**Affiliations:** a Federal Medical Center, Owerri, Nigeria; b van Horbachevsky Ternopil National Medical University, Ternopil, Ukraine; c Jiujiang University, Jiujiang, China; d Babcock University, Ilishan-Remo, Nigeria; e Babcock University Teaching Hospital, Ilishan-Remo, Nigeria; f Federal Medical Centre Bida, Bida, Nigeria; g Federal Medical Center, Jabi, Nigeria; h Federal Medical Centre, Abeokuta, Nigeria; i Windsor University School of Medicine, Cayon, St Kitts and Nevis; j University of Nigeria Teaching Hospital, Enugu, Nigeria; k Nnamdi Azikiwe University Teaching Hospital, Nnewi, Nigeria; l University of Ilorin Teaching Hospital, Ilorin, Nigeria; m Lakeshore Cancer Center, Lagos, Nigeria; n Lagos University Teaching Hospital, Lagos, Nigeria; o University of Ilorin, Ilorin, Nigeria; p University Hospital Lewisham, London, UK; q R-jolad Hospital, Gbagada, Nigeria; r The Manor Hospital Oxford, Oxford, UK; s Northwick Park Hospital, London North West NHS Trust, Harrow, UK; t Faculty of Clinical Sciences, College of Health Sciences, University of Ilorin, Ilorin, Nigeria; u Garkuwa Specialist Hospital, Kaduna, Nigeria.

**Keywords:** chronic kidney disease, comprehensive review, disease progression, management guidelines, treatment strategies

## Abstract

Chronic kidney disease (CKD) is a prevalent and progressive condition affecting millions worldwide. It is a long-term condition characterized by gradual loss of kidney function over time. The management of CKD is complex and requires a multidisciplinary approach. This review aims to outline the current management guidelines for CKD. The study included a comprehensive search of various PubMed, Embase, and the Cochrane Library databases for articles published between 2010 and 2023. The search terms used were "chronic kidney disease," "management," and "guidelines." The inclusion criteria were articles that provided management guidelines for patients with CKD. A total of 23 articles were included in the review. Most articles were based on the Kidney Disease Improving Global Outcomes guidelines, the most widely recognized and used guidelines for managing CKD. The study found that the guidelines emphasize the importance of early detection and management of CKD and the need for an approach that involves multiple disciplines in its management. The guidelines recommend several interventions to slow the progression of CKD, including blood pressure control, glycemic control in diabetic patients, and reduce proteinuria. Other interventions include lifestyle modifications such as dietary changes, physical activity, and smoking cessation. The guidelines also recommend regular monitoring of kidney function and referral to a nephrologist for patients with advanced CKD or other complications. Overall, the current management guidelines for CKD emphasize the importance of early detection and a multidisciplinary approach to its management.

## 1. Introduction

Chronic kidney disease (CKD) is a significant public health problem globally, affecting millions of people worldwide.^[1]^ The management of CKD is crucial to prevent the progression of the condition to end-stage renal disease (ESRD) and to reduce the risk of cardiovascular disease and other complications.^[1]^ Various management guidelines are available for CKD, which different organizations and expert panels develop.

A comprehensive review of the current management guidelines of CKD can provide valuable insights into the similarities and differences among these guidelines. It can help identify the best practices for managing CKD.^[2]^ Such a review can also help to identify gaps in the existing guidelines and areas where further research is needed to improve the management of CKD.

Several studies have comprehensively reviewed the current management guidelines for CKD – one study by Schena FP. (2011) analyzed the guidelines from 6 organizations and found considerable variability in the recommended management strategies for CKD.^[3]^ Another study by Ong et al^[4]^ reviewed the guidelines from 14 organizations and found that the recommendations were generally consistent. Still, there were variations in the evidence supporting the recommendations.

## 2. Objectives

The objectives of treatment for chronic kidney disease (CKD) are to slow down the progression of the disease, manage complications and symptoms, prevent or delay the commencement of end-stage kidney disease, and improve the patient’s quality of life, which requires dialysis or kidney transplantation for survival. To achieve these objectives, treatment may involve a combination of lifestyle modifications, medications, and medical procedures, such as:

### 2.1. Blood pressure control

High blood pressure is a common complication of CKD, and controlling it is essential in slowing the progression of the disease. Medications like angiotensin converting enzyme inhibitors and angiotensin receptor blockers are often prescribed to manage hypertension.

### 2.2. Blood sugar control

If the patient has diabetes, tight control of blood sugar levels can help to prevent or delay the onset of renal damage.

### 2.3. Dietary modifications

A diet low in salt, phosphorus, and protein may be recommended to reduce the kidney workload and manage symptoms.

### 2.4. Medications to treat complications

Medications may be prescribed to manage anemia, bone disease, and other CKD-related complications.

### 2.5. Dialysis or kidney transplant

In advanced stages of CKD, when the kidneys have lost most of their function, dialysis or kidney transplantation may be necessary to replace the kidney function and maintain the patient’s health.

## 3. Methodology

Millions of people are affected by CKD, a common and progressive condition. Successfully managing CKD is a complex process requiring a collaborative, multidisciplinary approach. Therefore, a review of current management guidelines for CKD was conducted to outline the best practices for CKD management.

### 3.1. Search strategy

The review included a comprehensive search of various PubMed, Embase, and the Cochrane Library databases for articles published up until 2022. The search terms used were “chronic kidney disease,” “management,” and “guidelines.” The inclusion criteria were articles that provided management guidelines for CKD patients.

### 3.2. Selection criteria

Two reviewers independently screened all titles and abstracts for eligibility after the initial search. The same 2 reviewers reviewed full texts of potentially relevant publications. A third reviewer resolved any disagreements. The inclusion criteria were articles that provided management guidelines for CKD patients.

### 3.3. Data extraction

Two reviewers collected data independently using a predefined data extraction form. The extracted data included the author’s name, year of publication, country of origin, CKD stage, interventions, and outcomes. A third reviewer resolved any disagreements.

### 3.4. Data analysis

The review identified 23 articles that met the inclusion criteria. Most articles were based on the Kidney Disease Improving Global Outcomes (KDIGO) guidelines, the most widely recognized and used guidelines for managing CKD. According to the study, the guidelines prioritize the timely detection and treatment of CKD and advocate for a collaborative approach involving multiple disciplines in its management.

### 3.5. Interventions

The guidelines recommend several interventions to slow the progression of CKD, including blood pressure control, glycemic control in diabetic patients, and reducing proteinuria. Other interventions include lifestyle modifications such as dietary changes, physical activity, and smoking cessation.

### 3.6. Limitations

The review identified some limitations of the current guidelines, such as the lack of consideration for specific patient populations, such as the elderly and those with comorbidities. Also, there is a need for more studies on the effectiveness of interventions and personalized management of CKD.

### 3.7. Conclusions

In conclusion, the current management guidelines for CKD emphasize the importance of early detection and a multidisciplinary approach to its management. Nevertheless, more research needs to be done on personalized management and consideration for specific patient populations. The findings of this comprehensive review help healthcare professionals better understand the current management guidelines for CKD and inform their practice.

## 4. Definition and epidemiology

Chronic renal disease is a global health problem affecting millions worldwide.^[1]^ The epidemiology of CKD is complex and influenced by various factors, including age, gender, race/ethnicity, and comorbidities.

The prevalence of CKD varies across different populations and is influenced by various factors such as age, gender, race/ethnicity, and comorbidities.^[5]^ In the United States, CKD affects around 15% of the adult population, with higher rates observed in older adults, African Americans, and Hispanics.^[5]^ In Asia, the prevalence of CKD is estimated to be around 10% to 15%, with a higher burden of disease in countries such as China and India.^[1]^

The incidence of CKD is also influenced by numerous factors such as age, gender, race/ethnicity, and comorbidities. In the United States, the incidence of ESRD due to CKD is highest among African Americans, followed by Hispanics, Native Americans, and Asians.^[5]^ In Asian countries such as Japan and South Korea, the incidence of ESRD is higher among older adults.^[6]^

CKD is a complex disease influenced by multiple risk factors, including diabetes, hypertension, obesity, smoking, and a family history of kidney disease. Hypertension and diabetes are the leading causes of CKD globally, accounting for around two-thirds of all cases.^[7]^ Other risk factors, e.g., obesity and smoking, have also been associated with a high risk of CKD.^[8]^

## 5. Pathophysiology

Chronic Kidney disease can arise from cell injury that may cause damage to a kidney section that may or may not have been directly impacted by an insult or toxin.^[9]^ It is most often an insidious process compared to its more acute counterpart, Acute Kidney disease.^[10]^

The kidneys have been observed to have a greater rate of blood flow than other well-perfused organs, and as such, they are more likely to be exposed to substances that may be toxic to them.^[11]^ The glomerular capillaries may also be prone to injury because glomerular filtration depends on high intra and trans-glomerular pressure; hence any disease process that raises this pressure may affect them negatively. Glomerular hypertension and hyperfiltration are 2 common culprits implicated in the development of CKD.^[9]^

The negatively charged molecules on the membrane of the glomerular filtration apparatus would generally act like a filter preventing the passage of anionic macromolecules, which would explain why some of those macromolecules in the urine would serve as an indication of glomerular dysfunction.^[9,10]^ Damage to a glomeruli, e.g., from hypoxia, can spread to the tubulointerstitial compartment causing injury or remodeling of the tissue because decreased blood flow either before or at the glomerulus can cause decreased peritubular blood flow and ultimately damage the tubulointerstitium.^[11]^

Glomerulosclerosis and tubulointerstitial fibrosis are often implicated in CKD of any underlying cause.^[9]^ Some biochemical pathways have been noted to contribute to the progression of CKD. Angiotensin II can induce TGF-β1, as well as other inflammatory mediators like connective tissue growth factor, leading to the compression and the consequent elaboration of the extracellular matrix; it can also induce oxidative stress, which can upregulate TGF-β1 leading to the propagation of a fibrosing response by inducing the formation of extracellular matrix.^[10,11]^ Tissue proteases that would typically degrade matrix proteins are inhibited.

Some of the more popular causes of renal injury are based on immunologic reactions, tissue hypoxia, ischemia, exogenic agents, e.g., drugs, endogenous substances, e.g., glucose in excess, genetic defects, etc.^[12]^

CKD’s pathophysiology can be due to a glomerular, tubular, or vascular injury that has occurred over time. Glomerular impairment can be due to a hereditary condition, e.g., as seen in Alport syndrome; it can also be acquired from an injury, toxin, or prolonged stress.^[9]^ An immune complex deposition is often associated with many Kidney diseases, and various disease conditions can occur depending on the location of the deposition, e.g., mesangial deposition as in Henoch Schonlein Purpura, Subendothelial deposition as in Membranoproliferative Glomerulonephritis Subepithelial deposits as in Membranous nephropathy, etc.^[11]^ When circulating inflammatory cells come in contact with these immunoglobulins, it forms a strong inflammatory reaction; these inflammatory cells can ultimately cause damage to the vascular walls and filtration barrier, causing even more recruitment of inflammatory cells and worsening kidney damage.^[10]^ Complement activation can also mediate tissue injury after immune complex deposition. Injury can also be non-immunologic glomerular, as in hemodynamic, metabolic, or toxic injury.^[12]^

Systemic and glomerular hypertension are other processes frequently implicated in CKD.^[9]^ It is seen when the blood pressure is high enough to exceed the protective autoregulation of the kidney, causing glomerular filtration barrier injury.^[13]^ In chronic hypertension, arteriolar vasoconstriction and sclerosis can lead to glomerular and tubulointerstitial atrophy, while glomerular hypertension will cause glomerulosclerosis; all these processes ultimately damage the kidneys.^[11]^

Tubulointerstitial impairment can also cause long-term renal impairment because their associated fibrosis results from inflammatory infiltrates that cause extracellular matrix accumulation.^[10]^ Fibrosis is also associated with proinflammatory, profibrotic, and vasoconstrictive factors that worsen kidney disease.

Hypoxia-inducible factor 1 is a factor that has been noted to stimulate the transition of epithelial cells into mesenchymal cells.^[9,11]^ This process has been associated with the worsening of fibrosis and renal failure. It is also related to the accumulation of extracellular matrix. Changes in the expression of this factor have been noted to correlate with the extent of tubulointerstitial damage.^[12]^

Proteinuria is also associated with damage to the tubulointerstitium due to direct toxicity to the tubules themselves, changes in the epithelial metabolism of the tubules, increased cytokine and chemokine synthesis, and increased expression of adhesion molecules.^[14]^

Other associated factors include decreased perfusion of the kidneys as in shock, smoking, chronic non-steroidal anti-inflammatory drugs use, congenital disabilities that affect the kidney, like the presence of posterior urethral valves, and aging-related changes.^[12,14]^

## 6. Laboratory abnormalities and diagnosis

CKD is a severe medical condition affecting millions worldwide. It is a common medical condition characterized by a progressive loss of renal function over time. The KDIGO defines CKD as abnormalities of kidney structure or function, present for more than 3 months, with health implications.^[15]^

Table 1 details the laboratory abnormalities and diagnosis of CKD.

**Table 1:** Details the laboratory abnormalities and diagnosis of chronic kidney disease.

*Laboratory abnormalities:* The kidneys primarily maintain homeostasis through their fundamental functions of filtration, reabsorption, and secretion. It plays a vital role in the regulation of extracellular fluid volume, serum osmolality, and electrolyte concentrations, excretion of waste products and toxins such as urea, creatinine, and uric acid, as well as the production of hormones, e.g., erythropoietin and 1,25 dihydroxy vitamin D and renin.^[16]^ CKD results in the derangement of the homeostatic mechanisms of the kidneys, which leads to fluid and electrolyte disturbance, endocrine and metabolic disorders, and hematological abnormalities. Consequently, the laboratory abnormalities in CKD include impaired glomerular filtration propagated by the Renin-Angiotensin System (RAS) activation, which clinically manifests as proteinuria.^[17]^ Fluid and electrolyte disturbances result in hyponatremia, hyperkalemia, hypervolemia, hyperphosphatemia, hyperchloremia, metabolic acidosis, and hypocalcemia. Endocrine and metabolic disorders of CKD comprise hyperuricemia, abnormal lipid metabolism (hypertriglyceridemia, decreased HDL levels), Vitamin D deficiency, and elevated parathyroid hormone levels. A classical manifestation of this is chronic kidney disease-mineral and bone disorder (CKD-MBD), a systemic disorder of mineral and bone metabolism with abnormalities of calcium, phosphorus, PTH, or vitamin D metabolism. Others include abnormalities in gonadal and growth hormones, causing subfertility and impaired growth.^[17,18]^ Hematological abnormalities include anemia, lymphocytopenia, leukopenia, and thrombocytopenia. Anaemia in CKD is usually normocytic normochromic; it arises from multiple factors such as reduced erythropoietin production, decreased life span of red blood cells, and impaired intestinal iron absorption.
*Diagnosis:* The primary biochemical abnormalities required for the diagnosis of CKD are reduced GFR and proteinuria (albuminuria). The clinical evaluation of individuals suspected of CKD should be targeted at confirming the diagnosis and making an etiological diagnosis to identify the underlying medical condition. KDIGO guideline requires the persistence or progression of detected abnormality for ≥ 3 mo.^[15]^ Evaluating glomerular filtration rate using serum creatinine and a GFR estimating equation such as the CKD-EPI (Chronic Kidney Disease Epidemiology Collaboration) and the Modified Diet in Renal Disease (MDRD) equation. The CKD-EPI equation is preferred due to higher precision and accuracy.^[19]^ Diagnosis of CKD based on reduced eGFR requires 2 measurements of eGFR < 60 mL/min/1.73 m^2^ over 3 months.^[17]^ Further tests such as serum Cystatin C and creatinine clearance are recommended to confirm CKD diagnosis where serum creatinine-based eGFR is deemed unreliable. Cystatin C-based eGFR (eGFRcys) measuring serum Cystatin C is recommended for diagnosis confirmation in adults with isolated reduced eGFRcreat (45–59 mL/min/1.73 m^2^) without markers of kidney damage.^[15]^ The combined creatinine–cystatin C equation has higher precision and accuracy for GFR estimation than individual equations, providing a more accurate classification of CKD.^[20]^ Albuminuria is characterized by the abnormal presence of albumin in the urine. It is defined as an albumin excretion rate ≥ 30 mg per 24 h, ACR of ≥ 30 mg/g (3 mg/mmol). Urine albumin: creatinine ratio (ACR) is preferred over protein: creatinine ratio (PCR) for detecting low levels of proteinuria due to higher sensitivity. Confirmed ACR ≥ 3mg/mmol is termed clinically significant proteinuria. ACR levels between 3 to 70 mg/mmol should be re-checked and verified using an early morning urine sample.^[21]^ Urinalysis is necessary for the assessment of hematuria. Hematuria 1 + should be investigated further.^[21]^ Aetiological diagnosis is essential for the evaluation of the underlying cause of CKD. These tests include kidney biopsy, renal imaging with an ultrasound scan, CT scan, and MRI, and genetic testing indicated in certain conditions. KDIGO recommends that CKD is classified based on cause, GFR category, and albuminuria category (CGA). This is crucial for prognostication; decreasing eGFR and increasing proteinuria are independently associated with a poorer prognosis of chronic kidney disease.^[21]^

Overall, the diagnosis and management of CKD rely on assessing various laboratory parameters, including serum creatinine, estimated glomerular filtration rate (eGFR), and albuminuria. However, early diagnosis and management of CKD are crucial to prevent the disease’s progression and reduce the risk of associated complications, such as cardiovascular disease, anemia, and bone disease.

Laboratory abnormalities play a critical role in the diagnosis and management of CKD. Standard laboratory tests used to diagnose CKD include serum creatinine, estimated glomerular filtration rate (eGFR), and urine albumin-creatinine ratio (ACR).

According to the current management guidelines of CKD, a diagnosis of CKD is made when there is evidence of kidney damage or a reduction in kidney function for more than 3 months.^[15]^ Kidney damage is defined as albuminuria, hematuria, structural abnormalities, or a history of kidney transplantation. A reduction in kidney function is defined as an eGFR of below 60 mL/min/1.73 m2 for more than 3 months.

Serum creatinine is a waste product produced by muscle metabolism and excreted by the kidneys. Elevated serum creatinine levels indicate impaired kidney function and are used to estimate the eGFR, which measures the rate at which the kidneys filter blood. A reduced eGFR is a crucial indicator of CKD.

Serum creatinine is the commonly used laboratory parameter for estimating kidney function. However, its accuracy is affected by various factors such as age, gender, and muscle mass. To address these limitations, eGFR is calculated using equations considering the patient’s age, gender, race, and serum creatinine level. The Modification of Diet in Renal Disease (MDRD) equation is the most commonly used equation.^[15]^

Albuminuria is an important marker of kidney damage in CKD. It is a urinary albumin-to-creatinine ratio (ACR) of 30 mg/g or higher. Sometimes, a 24-hour urine collection is used to measure albumin excretion.^[15]^ Elevated levels of ACR are indicative of kidney damage and are used to monitor the progression of CKD.

In addition to these tests, other laboratory abnormalities may be observed in patients with CKD. These include electrolyte imbalances (such as hyperkalemia and hypocalcemia), anemia, abnormalities in parathyroid hormone (PTH) levels, and phosphate metabolism. The diagnosis and management of CKD are complex and require a multidisciplinary approach. Clinical guidelines provide a framework for healthcare professionals to provide optimal care for patients with CKD. A comprehensive review of current management guidelines for CKD would be essential in determining the most up-to-date and evidence-based approaches for diagnosing and managing CKD. This would include recommendations for laboratory testing and other diagnostic and therapeutic interventions to prevent the progression of the disease and reduce the risk of associated complications. Imaging tests like ultrasound and computerized tomography (CT) scans may also assess the kidneys’ size and shape.

CKD = chronic kidney disease, KDIGO = kidney disease improving global outcome, PTH = parathyroid hormone.

## 7. Treatment

The latest guidelines for managing CKD are provided by the KDIGO organization. These guidelines were last updated in 2020.^[22]^ The key recommendations for managing CKD from KDIGO include the following:

Accurate and timely diagnosis and staging of CKD using the Kidney Disease Outcome Quality Initiative (KDOQI) guidelines or KDIGO guidelines.

Identifying and managing risk factors for CKD progression, including blood pressure control, glycemic control in diabetes, lipid management, smoking cessation, and weight management.

Regularly monitor kidney function, proteinuria, blood pressure, and other risk factors for CKD progression.

Use of renin-angiotensin-aldosterone system (RAAS) blockade in patients with albuminuria and hypertension unless contraindicated.

Use non-steroidal anti-inflammatory drugs cautiously in CKD patients and avoid nephrotoxic agents whenever possible.

Evaluation and management of CKD-associated complications, including anemia, mineral and bone disorders, cardiovascular disease, and infectious diseases.

Education and counseling of patients with CKD to promote self-management and adherence to treatment.

Referral to a nephrologist when appropriate, based on the patient’s CKD stage and progression risk.

These guidelines provide a comprehensive approach to managing CKD and can be used to improve patient outcomes and quality of life. Table 2 gives more information on managing risk factors for CKD progression.

**Table 2:** Gives more information on managing risk factors for CKD progression.

**Blood pressure control:** An ACEI or ARB should be the first-line agent for antihypertensive therapy for CKD patients and is recommended for patients with albuminuria regardless of the need for blood pressure control. Renin-angiotensin system inhibitors also have specific reno-protective effects in proteinuric non-diabetic chronic renal failure independent of blood pressure control, reducing proteinuria, and chronic kidney disease progression as defined by doubling baseline serum creatinine or development of end-stage kidney disease. The effect is most significant in those with higher levels of proteinuria.^[22]^ The indications for starting renin-angiotensin system antagonists in chronic kidney disease are summarized below: Diabetes and ACR ≥ 3 mg/mmol. Hypertension and ACR ≥ 30 mg/mmol ACR ≥ 70 mg/mmol irrespective of hypertension or cardiovascular disease. ACR – Albumin to creatinine ratio. Blood pressure targets in chronic kidney disease are shown below: CKD: BP 120–139/<90 mm Hg. CKD and diabetes mellitus: BP 120–129/<80 mm Hg. CKD and ACR ≥ 70 mg/mmol: BP 120–129/<80 mm Hg. ACR = albumin to creatinine ratio, BP = blood pressure, CKD = chronic kidney disease. Potassium and eGFR should be measured before beginning renin-angiotensin system inhibitors and repeated 1–2 wk after starting renin-angiotensin system inhibitors and after each dose increase. Renin-angiotensin system antagonists should not be routinely given to people with chronic renal disease if the pretreatment potassium is more than 5.0 mmol/L and stopped if the potassium increases to ≥ 6.0 mmol/L and other drugs are known to promote hyperkalemia. Its blockade could lead to acute renal failure in conditions such as bilateral renal artery stenosis, where angiotensin is critical in preserving the intraglomerular pressure and GFR. Thus, checking serum creatinine and potassium about 1–2 weeks after starting or changing the dose of angiotensin-converting enzyme (ACE) inhibitor and angiotensin II receptor blocker (ARB) is recommended.
**Diabetes mellitus:** One in every 2 people who visit their primary care practice with type 2 diabetes will have CKD, diabetes is a significant risk factor for CKD, with up to 40% of CKD being caused by diabetes.^[23]^ The presence of diabetes worsens the outcomes in all stages of CKD (cardiovascular outcomes, dialysis survival, and post-transplant survival).^[23]^ The relatively strict control of blood glucose (hemoglobin A1c ≤ 7%) in type 1 and type 2 diabetes decreases the development of diabetic nephropathy and its progression. Diligent blood pressure control reduces kidney disease progression and cardiovascular morbidity and mortality among people with diabetes. Diabetes treatment targets: ≤7% (range 6.5–7.5); it needs individualization according to patient circumstances (e.g., disease duration, life expectancy, significant comorbidities, and established vascular complications). Interpret with caution if hemoglobin (Hb) is changing. The FDA revised its guidance for using metformin in CKD in 2016, recommending using eGFR rather than serum creatinine to guide treatment and expanding the pool of people with kidney disease for whom metformin treatment should be considered.^[24]^ The revised FDA guidance states that: Metformin is not indicated in patients with an eGFR < 30 mL/min/1.73 m^2^. eGFR should be monitored when taking metformin. When the eGFR decreases to less than 45 mL/min/1.73 m^2^, the benefits and risks of continuing treatment should be reevaluated.^[25]^ Patients with an eGFR of less than 45 mL/min/1.73 m^2^. Should not be given metformin. Metformin should be temporarily discontinued during or before iodinated contrast imaging examinations in patients with eGFR 30–60 mL/min/1.73 m^2^. SGLT2 inhibitors are recommended for people with stage 3 CKD or higher and type 2 diabetes, as they slow CKD progression and reduce heart failure risk independent of glucose management.^[26]^ GLP-1 receptor agonists are suggested for cardiovascular risk reduction if such risk is a predominant problem. They reduce risks of cardiovascular disease events and hypoglycemia and appear to slow CKD progression, possibly.^[27]^ Preferred antihypertensive therapy among people with diabetes with hypertension is with an ACEI or an ARB. ACEI or ARB therapy is also recommended for normotensive diabetics with microalbuminuria.
**Cardiovascular disease:** Cardiovascular disease is the leading cause of morbidity and mortality among patients with CKD. Recent studies have demonstrated that even early-stage chronic kidney disease constitutes a significant risk factor for cardiovascular events and death. Similarly, cardiovascular disease is a risk factor for the progression of CKD. Statins decrease the relative risk of cardiovascular events to a similar extent among people with and without CKD. However, the benefit is more significant in patients suffering from CKD because of the greater baseline risk for patients with chronic kidney disease. In addition to reducing cardiovascular risk, drugs like statins may have a role in preventing the progression of renal disease and reducing albuminuria. However, evidence for these outcomes is less robust. Based on the most recent KDIGO (Kidney Disease Improving Global Outcomes) guideline, statin use is: Recommended for all non-dialysis CKD patients ≥ 50 years of age, regardless of the stage of disease or the presence or absence of albuminuria. Suggested for non-dialysis or non-kidney transplant CKD patients who are 18 to 49 years of age and have an estimated risk of > 10% for a 10-year incidence of coronary death, or non-fatal myocardial infarction (includes any with coronary disease, diabetes mellitus, or ischemic stroke). Suggested for all kidney transplant patients, regardless of age. Suggested to continue in patients already receiving statins at dialysis initiation. Suggested not to be initiated in patients with dialysis-dependent CKD.^[28]^
**Comorbidities:** Patients suffering from CKD are at increased risk of developing several comorbidities, or co-existing health conditions, which can impact their overall health outcomes. Here are some of the most common comorbidities of CKD: *Cardiovascular disease*: CKD is strongly associated with an elevated risk of cardiovascular disease, including heart attacks, strokes, and heart failure. This is because the kidneys play a crucial role in regulating blood pressure and removing excess fluid and waste products from the body, and when they are not functioning correctly, this can strain the heart and blood vessels.^[19]^ *Diabetes*: Diabetes is a leading cause of chronic kidney disease; the 2 conditions often co-exist. Individuals with diabetes are more likely to develop kidney disease, and people with CKD are more likely to develop diabetes. High blood sugar levels can damage the kidneys over time.^[20]^ *Anemia*: Anemia is a common complication of CKD. It occurs when the kidneys can no longer produce enough erythropoietin hormone, which stimulates red blood cell production. This can result in fatigue, weakness, and shortness of breath.^[21]^ *Bone disease*: CKD can also lead to renal osteodystrophy, a group of bone disorders resulting from the kidneys’ inability to regulate calcium and phosphorus levels in the blood. This can cause weakened bones, bone pain, and an increased risk of fractures.^[22]^ *Hyperkalemia*: Hyperkalemia is a common complication of chronic kidney disease (CKD) and is considered a comorbidity of CKD. CKD can lead to a decreased ability of the kidneys to eliminate excess potassium from the body, resulting in high levels of potassium in the blood, known as hyperkalemia. Hyperkalemia can cause severe symptoms, including muscle weakness, heart palpitations, and cardiac arrest. Individuals with CKD must manage their potassium levels through diet, medications, and close monitoring by healthcare.^[23]^ *Metabolic acidosis*: Metabolic acidosis is a common complication of chronic kidney disease (CKD) and is considered a comorbidity of CKD. The kidneys play a crucial role in maintaining the acid-base balance in the body by regulating the levels of bicarbonate, a base, and hydrogen ions, an acid. In CKD, the kidneys cannot excrete enough acid or produce enough bicarbonate, leading to a buildup of acid in the blood and decreased bicarbonate levels.^[24]^ This results in a condition known as metabolic acidosis. Metabolic acidosis can cause various symptoms, including fatigue, shortness of breath, and confusion, and can lead to complications such as bone disease, muscle wasting, and kidney damage. Treatment for metabolic acidosis in CKD typically involves addressing the underlying cause, such as managing kidney function and providing bicarbonate supplements. Individuals with CKD must regularly monitor their acid-base balance to prevent complications associated with metabolic acidosis.^[24]^ *Dyslipidemia and hypercholesterolemia*: Dyslipidemia is a condition characterized by abnormal levels of lipids (fats) in the blood, including high levels of LDL cholesterol (the “bad” cholesterol) and low levels of HDL cholesterol (the “good” cholesterol). In people with CKD, dyslipidemia is often present due to the decreased ability of the kidneys to remove lipids from the blood.^[25]^ Hypercholesterolemia is a condition with an abnormally high cholesterol level in the blood, including high LDL cholesterol levels.^[26]^ This can lead to atherosclerosis (narrowing the arteries due to the buildup of cholesterol and other substances), increasing the risk of heart disease and stroke. Both dyslipidemia and hypercholesterolemia are common in people with CKD. They can contribute to the increased risk of cardiovascular disease, a significant cause of morbidity and mortality in this population. Therefore, managing these conditions is integral to managing CKD.^[26]^ *Malnutrition*: CKD can also lead to malnutrition, as the kidneys filter waste products from the blood and excrete them in the urine. When the kidneys are not functioning properly, waste products can build up in the blood, leading to a loss of appetite, nausea, and vomiting.^[27]^ *Depression and anxiety*: People with CKD may also experience depression and anxiety, as the condition can significantly impact their quality of life. They may have to make significant lifestyle changes, such as following a restricted diet and undergoing regular dialysis treatments, which can be stressful and challenging to manage.^[28]^ Overall, the comorbidities of CKD can significantly impact a patient’s health outcomes and quality of life. Therefore, it is essential to manage these conditions in addition to treating CKD itself.^[29]^
**Dialysis and RRT options:** Dialysis and renal replacement therapy (RRT) are 2 options for individuals with advanced stages of CKD. Dialysis is a medical procedure that helps filter waste and excess fluids from the blood when the kidneys can no longer perform this function adequately.^[30]^ There are 2 types of dialysis: hemodialysis and peritoneal dialysis. Hemodialysis involves the use of a machine called a dialysis machine to filter the blood. During the procedure, the patient’s blood is drawn from an artery in the arm or leg and passed through a filter in the machine.^[29]^ The vein returns the filtered blood to the patient’s body On the other hand, peritoneal dialysis involves using the lining of the patient’s abdomen as a filter. During the procedure, a catheter is inserted into the patient’s abdomen, and a special fluid called dialysate is infused into the abdomen. The dialysate pulls waste products and excess fluid from the blood into the abdominal cavity. After several hours, the dialysate is drained from the abdomen, taking the waste products and excess fluids.^[28]^ Renal replacement therapy (RRT) is an option for individuals with advanced CKD who can no longer undergo dialysis. It involves using a kidney transplant to replace the patient’s damaged kidneys with a healthy donor kidney.^[30]^ Both dialysis and RRT can significantly improve the quality of life for individuals with advanced CKD. However, they are not without risks and complications, and the decision to undergo either treatment should be made in consultation with a healthcare professional.^[31]^

Chronic kidney disease is an increasingly common clinical problem that raises a patient’s risk for developing several life-threatening medical conditions, including end-stage renal disease (ESRD) and cardiovascular disease. Appropriate treatment can delay or prevent these adverse outcomes.^[30,31]^ The direct management of chronic renal failure focuses on renin angiotensin aldosterone system antagonist (RAAS) and blood pressure control. Management also comprises optimal management of common comorbid conditions, e.g., diabetes, and addressing cardiovascular risk factors to decrease the risk for CVD. Also essential are patient education and a multidisciplinary approach to disease management that include dieticians, social workers, and other health care providers.^[31]^

## 8. Conclusion

CKD is a progressive and irreversible condition that can lead to ESRD requiring dialysis or kidney transplantation. Over the years, various management guidelines have been developed to optimize care for patients with CKD and reduce the risk of complications.

The current management guidelines for CKD recommend early detection and intervention to slow or halt the progression of the disease. This involves regular monitoring of kidney function, blood pressure control, and lifestyle modifications, such as a healthy diet, regular exercise, and smoking cessation. The guidelines also recommend using medications to manage complications such as anemia, bone disease, and cardiovascular disease.

Additionally, the guidelines highlight the importance of a multidisciplinary approach to managing CKD. This involves collaboration between nephrologists, primary care physicians, dietitians, pharmacists, and other healthcare professionals to provide comprehensive and coordinated care. With proper management and timely intervention, it is attainable to slow or halt the progression of CKD and improve outcomes for patients with this condition.

## Author contributions

**Conceptualization:** Chukwuka Elendu, Rhoda C. Elendu, Joy M. Enyong, Chinazo J. Okenwa.

**Data curation:** Ibukunoluwa V. Ishola, Kehinde A. Opashola, John O. Fatoye.

**Formal analysis:** Ozigis O. Mutalib.

**Investigation:** Chukwuka Elendu.

**Methodology:** Joy M. Enyong, Chinazo J. Okenwa.

**Project administration:** Emmanuel S. Meribole, Kehinde A. Opashola.

**Resources:** Emmanuel O. Egbunu, Chinazo J. Okenwa, Ewezugachukwu D. Umeh, Ozigis O. Mutalib.

**Software:** Emmanuel O. Egbunu.

**Supervision:** Chukwuka Elendu, Rhoda C. Elendu.

**Validation:** Joy M. Enyong, Chinazo J. Okenwa.

**Visualization:** Chukwuka Elendu, Rhoda C. Elendu, Emmanuel O. Egbunu.

**Writing – original draft:** Chukwuka Elendu, Rhoda C. Elendu, Joy M. Enyong, Ozigis O. Mutalib, Kehinde A. Opashola.

**Writing.–.review & editing:** Jennifer O. Ibhiedu, Ibukunoluwa V. Ishola, Emmanuel S. Meribole, Sodiq O. Lawal, Geraldine C. Okafor, Ewezugachukwu D. Umeh, John O. Fatoye, Temitope I. Awotoye, Jewel I. Tobih-Ojeanelo, Habiba I. Ramon-Yusuf, Abiola Olanrewaju, Rechner N. Afuh, Joseph Adenikinju, Opeyemi Amosu, Abdulgafar Yusuf.
